# The Effects of Distancing Design Collaboration Necessitated by COVID-19 on Brain Synchrony in Teams Compared to Co-Located Design Collaboration: A Preliminary Study

**DOI:** 10.3390/brainsci14010060

**Published:** 2024-01-07

**Authors:** Yi-Teng Shih, Luqian Wang, Clive H. Y. Wong, Emily L. L. Sin, Matthias Rauterberg, Zhen Yuan, Leanne Chang

**Affiliations:** 1 School of Design, The Hong Kong Polytechnic University, Hong Kong; 2 Department of Psychology, The Education University of Hong Kong, Hong Kong; 3 Department of Industrial Design, Eindhoven University of Technology, 5612 AZ Eindhoven, The Netherlands; 4 Faculty of Health Sciences, University of Macau, Macau; 5 School of Communication, Hong Kong Baptist University, Hong Kong

**Keywords:** collaborative design, inter-brain synchrony (IBS), hyper-scanning, design cognition, COVID-19

## Abstract

Due to the widespread involvement of distributed collaboration triggered by COVID-19, it has become a new trend that has continued into the post-pandemic era. This study investigated collective performance within two collaborative environments (co-located and distancing settings) by assessing inter-brain synchrony patterns (IBS) among design collaborators using functional near-infrared spectroscopy. The preliminary study was conducted with three dyads who possessed 2–3 years of professional product design experience. Each dyad completed two designated design tasks in distinct settings. In the distributed condition, participants interacted through video conferencing in which they were allowed to communicate by verbalization and sketching using a shared digital whiteboard. To prevent the influences of different sketching tools on design outputs, we employed digital sketching for both environments. The interactions between collaborators were identified in three behaviors: verbal only, sketch only, and mixed communication (verbal and sketch). The consequences revealed a higher level of IBS when mixed communication took place in distributed conditions than in co-located conditions. Comparably, the occurrence of IBS increased when participants solely utilized sketching as the interaction approach within the co-located setting. A mixed communication method combining verbalization and sketching might lead to more coordinated cognitive processes when in physical isolation. Design collaborators are inclined to adjust their interaction behaviors in order to adapt to different design environments, strengthen the exchange of ideas, and construct design consensus. Overall, the present paper discussed the performance of virtual collaborative design based on a neurocognitive perspective, contributing valuable insights for the future intervention design that promotes effective virtual teamwork.

## 1. Introduction

Teamwork innovation has long been recognized as a core competitiveness in an organization’s ability to address complex problems. Social distancing restrictions during the COVID-19 pandemic have enforced traditional co-located collaborative mode into virtual teamwork in an accelerative way, resulting in great transformation for design collaborators that largely influence their way of communicating and interacting. To adapt to this COVID-19-related disruption, organizations were striving to embrace information and communication technologies (ICTs), such as video conferencing platforms, web-based tools, and computer-aided systems, in order to facilitate efficient virtual teamwork. However, the surge in ICT utilization poses a major challenge to the digital resilience of both individuals and organizations. Previous studies [1,2,3,4,5] have argued the impairments in collaborators’ cognition and communication within distributed collaborative design processes, leading to a deterioration of group performance. Researchers generally recognized that distributed teams reduced the awareness of collaborators [6,7,8] and the abundance of information [9], as well as aggravated miscommunication and conflicts [10,11]. Although it is widely accepted that co-located teams outperformed virtual teams, several studies suggested minor or insignificant correlations between teamwork design processes and collaborative (distributed or co-located) environments [12,13,14].

A considerable body of the literature has explored the implications of distributed design collaboration for design outcomes, collaboration efficiency, and overall group performance. Although several researchers have started to examine the effects of online collaboration on design activities, the predominant research methods employed continue to be self-reported questionnaires, interviews, and observations. Such traditional research methods are deficient in explaining the underlying factors that affect group design activities and interactions between design partners in different types of environments: co-located and distributed settings. Therefore, there is a need to gain insight into the neural activities and inter-brain connectivity during collaborative design processes, which showcases the potential to offer more objective evidence and demystify how team interactivity operates in various contexts.

A new technology from cognitive neuroscience, termed hyper scanning, has been developed and widely utilized to investigate inter-brain synchrony (IBS), a potential indicator for collective performance among teams [15,16]. IBS refers to the degree to which the brains of two or more individuals are synchronized. Reinero and colleagues [17] suggested that IBS can be a complementary approach for understanding collective performance among teams where self-report surveys are limited to capture design behavior. Another study, conducted by Lu et al. [18], examined the occurrence of IBS during collaborative tasks and interactive activities over time and observed a positive association between collaborative behavior and IBS. However, most hyper-scanning studies of interacting individuals are conducted in a face-to-face situation in the same room, where subjects can communicate mutually based on both verbal cues and non-verbal cues, such as facial expressions and body movements. A limited hyper-scanning study explores the effects of different collaborative environments on the degree of IBS, thereby impacting communication effectiveness and collective performance. Additionally, meager research focuses on design-related collaborations, which is a dynamic process involving various design behaviors to formulate design requirements, build design goals, and construct design solutions jointly. Only one relevant study [19] focused on the real-life creative problem-solving processes among teams, which is yet merely focused on the measurement of the left hemisphere of the brain.

In this study, we aimed to address three research questions. Firstly, we examined the design activities and interactions that occur in two distinct collaborative environments, co-located and distributed settings. Next, we explored the similarities and differences in IBS patterns when multiple design partners engage in design problem-solving processes within these two types of environments. Lastly, we investigated the correlations between the design collaboration environments and brain synchrony patterns, which in turn influence the design outputs and team performance. This study has the potential to unravel the neural underpinnings affecting design collaboration and its correlations with collective performance, as well as contribute new insights into the intervention design that promotes effective virtual teamwork, both in the context of design education and design practices.

## 2. Literature Review

### 2.1. Distributed Design Collaboration and Digital Resilience

The concept of collaborative design, as presented by Lahti et al. [20], entails an interactive and cooperative process in which participants engage in active communication to collectively establish a design objective, explore problem and solution spaces, and construct design solutions. Establishing effective communication between interactive individuals to exchange ideas during the concept generation process from diverse perspectives is a key element of a successful design collaboration driving product innovation [21]. The rapid development of the pandemic has forced designers to adapt to virtual teamwork; all design collaborations take place remotely using online video conferencing platforms, which has accumulatively become a trend that may continue during the post-pandemic era. Thus, design practitioners are required to increase their competencies of resilience to integrate technology into the collaborative experience so as to increase remote working benefits and mitigate digital stressors [22]. Digital stressors are commonly defined as any adverse effects that technology may have on users. Resilience refers to a process that enables people to effectively navigate and manage stressors, allowing them to bounce back from adversity [23]. The term digital resilience describes specific knowledge, skills, attitudes, competencies, and behaviors that individuals must acquire so that digital stressors can be counteracted. In this study, we defined digital resilience as the ability of collaborators to overcome technical difficulties and continuously adapt to online collaboration, even achieving collaborative effectiveness and design outcomes comparable to that of co-located collaboration.

Effective communication in design collaborations is featured by real-time interactions involving verbalization and the utilization of various visual techniques. In terms of the influences of virtual collaboration on design tasks, a variety of prior studies observed the overperformance of co-located collaborations compared to distributed teams. Based on the consequences of Liska’s research [24], virtual teams required approximately one-third (33.32%) more time to address the same assigned works and encountered a higher incidence of revising their solutions compared to co-located teams. Moreover, Hammond et al. [25] pointed out that even though design collaborators spend more time on the assignment, fewer design alternatives were delivered within such a distributed collaboration process. In addition, distributed collaboration can even induce specific interactive behaviors, as Kvan [26] and Lee & Do [27] propose, designers are prone to compromise in design decisions and showcase less willingness to explore the best solutions within virtual collaborative settings. Likewise, in another analysis [28], distributed collaborators were observed to exhibit a lower inclination towards using gestures, allocate more time towards sketching, and participate in fewer studies and discussions with respect to design problems. Several protocol studies [12,14] indicated no significant differences or even better performance in quality or novelty of design solutions within distancing cooperation. In addition, Yang et al. [29] found that in the context of online design collaboration, students tend to allocate more time to sketching compared to the co-present design environment. However, contrary to previous research, the researchers revealed a positive impact whereby increased sketching behaviors reduced cognitive load for students, facilitated the better expression of ideas, and promoted mutual understanding among interactive individuals.

Protocol studies, retrospective reviews, and observations alone are insufficient to explore how different collaboration environments impact the interaction behavior and collective performance of designers. Moreover, there is a lack of effective research on whether the changes in design behavior result in a weakening or compensating effect on collaborative performance. Therefore, in this study, we investigated the relationship between design collaborative behavior and collaborative performance from a brain-based perspective, focusing on brain movements and connectivity and brain synchrony, in various collaborative settings.

### 2.2. IBS and Brain Regions Relevant to Design Activities

Neuroimaging technology is a widely utilized technique that can capture brain information of interactive individuals within a non-invasive manner, thereby contributing to the study regarding interpersonal social interactions. However, due to the prior related works studying neurocognition that have focused on isolated individuals, the enigmatic box regarding how the brain engages in dynamic group collaborations has failed to be fully unraveled. An emerging technique termed hyper-scanning has been devised to concurrently capture and measure brain activations of multiple collaborative individuals [30]. Compared to conventional neuroimaging study designs [31], hyper-scanning experiments provide a more realistic approximation of interactions between individuals. The degree of IBS, the coordination of brain activity among collaborators, can be measured by the hyper-scanning method. IBS serves as a neuro mechanism that aids scientists in identifying brain regional connectivity and dynamics during social interaction tasks. Functional Near-infrared Spectroscopy (fNIRS) and Electroencephalography (EEG) have been more frequently applied than functional magnetic resonance imaging (fMRI) for measuring IBS, due to their reasonable spatial resolution, greater resilience to body movements and less limitation of experimental setting. As a result, fNIRS and EEG are arguably more suitable for studying IBS within naturalistic interactive environments [32,33].

Numerous studies have generally observed that IBS could be an objective and reliable indicator of collective performance. For instance, the occurrence of IBS often increases when team members communicate or infer intentions mutually [34]. Another study also observed a close relationship between group identification and IBS when individuals worked together to complete problem-solving tasks [17]. Likewise, a study carried out by Hsu et al. [35] revealed a stronger IBS among subjects in cooperative mode compared to single-player mode. Moreover, there was a noticeable decrease in the strength of IBS when subjects switched from being collaborators to competitors.

To the best of our knowledge, the majority of hyper-scanning studies examining interactions among individuals are always conducted in a co-located environment, featured by sufficient verbal and non-verbal cues. Merely a limited number of studies have investigated group interactions in distributed collaborative settings, where individuals exert greater efforts in deducing and predicting partners’ intentions. One EEG study undertook an experiment in which each pair of participants collaboratively played an online car racing game within a physically isolated environment [36]. The researchers found significant positive relations between better collective performance and increased brain synchrony. Another study also illustrated that face-to-face conditions promoted more cooperation and a higher IBS compared to face-blocked interactions. However, the tasks conducted in the above studies are a far cry from real-world collaboration and team interactions. Scientists still know little about temporal brain dynamics and how different cooperative environments affect IBS and collective performance among real-world teamworks.

Additionally, to date, very few research studies have studied real design collaborations from the neurocognition perspective. In design teamwork, team members often utilize communication via various ways for idea exchange and mutual understanding establishment, especially in problem-solving and concept-generation processes. One of the fNIRS-based hyper-scanning studies examined real-world creative problem-solving processes in teams and explored the temporal changes in IBS over time [19]. The main limitation of this study is that they restricted their study to measurements of the left hemisphere of the brain. The previous literature has shown that multiple areas of the brain are activated when performing activities similar to those design tasks, especially the prefrontal cortex (PFC) area [37,38]. The PFC is associated with multiple cognitive processes, including but not limited to planning, maintaining focus, information filtering, and executive function [39]. Within the realm of design creative tasks, the PFC plays a crucial role in various cognitive functions. Specifically, the PFC on the right is often involved in divergent thinking, while the opposite hemisphere is more active in rule-based design, goal-oriented planning, and analytical judgment [40]. Strong synchrony observed in the right PFC is linked to an increased level of ingenuity in generated solutions [41].

Furthermore, during the execution of creative tasks, the left and right dorsolateral prefrontal cortical areas (DLPFC) are both active [42]. Increased activation in the right DLPFC is typically associated with the performance of creative problem-solving and visual–spatial thinking [43]. The left DLPFC is also involved in creative tasks and exhibits greater activation when engaged in goal-oriented planning for innovative solutions. In addition, the right ventrolateral PFC (VLPFC) contributes to evaluating problems instead of solving problems, aiding in generating alternative assumptions in the problem space search [44]. By employing neuroscience methodologies to investigate design cognition, we can enhance comprehension of the neurocognitive processes associated with design and refine design thinking theory [45].

## 3. Research Methodology

This study investigated how distributed design collaborations impact design collaboration behaviors, as well as the associations between specific design activities and underlying neural activities involving IBS as a critical predictor. In light of the aims of this study, think-aloud protocol analysis was employed to analyze and identify design interaction behaviors into three interactive behaviors: verbal only, sketch only, and mixed communication (a combination of verbalization and sketching). According to these three design interaction approaches, recorded video data were segmented into smaller episodes, which are used as critical timecodes for subsequent brain activity segmentation and brain-to-brain synchrony analysis. Hence, this study commonly consists of five components: (i) experiment settings, (ii) data collection, (iii) interaction segmentation, (iv) brain activity segmentation, and (v) inter-brain connectivity analysis.

### 3.1. Participants

The preliminary study was conducted with three dyads of volunteers (1 female–female, 1 male–male, and 1 female–male) who were equipped with 2–3 years of professional product design experience. All subjects self-identified as right-handed, healthy, and reported no visual impairments or neurological conditions. The age range of participants varied between 22 and 25 years (Mean = 23.3, SD = 0.943). Participants paired in the same dyad were previously acquainted, so that they could conduct the design process quickly and smoothly after a warm-up session. Informed consent was obtained from both dyad members prior to participation. The overarching aim was to design a paradigm that closely resembled real-world design collaboration scenarios. Therefore, dyads were asked to work on design problems for a continuous time of 25–30 min with little instruction and no interventions. All dyads received consistent design briefs and instructions. Ethical approval was obtained for this project on 14 September 2021 (approval number: HSEARS20210914003).

### 3.2. Experimental Settings and Procedures

**Figure 1:**
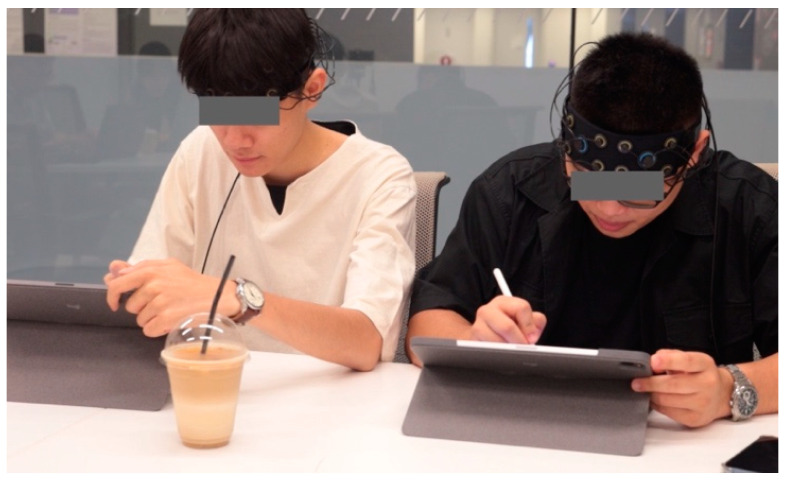
Co-located design collaboration: each dyad was seated together on the same side of a rectangular table within the same room.

**Figure 2:**
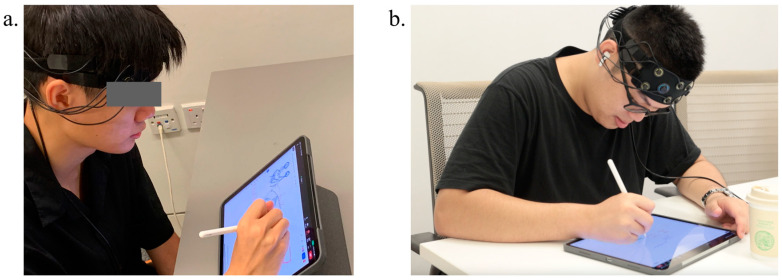
Distributed design collaboration, two subjects were situated in separate rooms. (**a**) The view from camera A; (**b**) the view from camera B.

**Figure 3:**
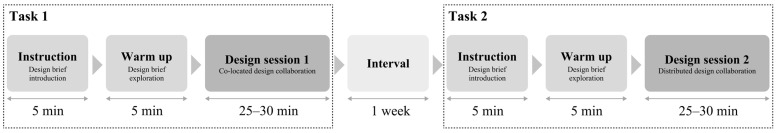
Sequence and duration of experimental sessions.

This study was conducted in carefully configured design studio spaces in order to create a controlled environment that is as close to a real-world setting as possible. The experimental procedure includes two tasks, requiring participants to undertake two separate conceptual design tasks within different design collaboration environments: co-located and distributed. In terms of task 1, each pair of participants was seated together on the same side of a rectangular table within the same room (see Figure 1), and a fNIRS cap was fitted over the forehead of each participant. After subjects filled in the consent form, the design brief was provided and elaborated to the participants prior to the start of the experiment. Dyads were asked to work together to design a toy and collaboratively define the target groups and contexts. Participants were then provided with a five-minute warm-up session for a brief discussion to determine their specific design scope. No fNIRS scanning happened during the warm-up session. Subsequently, a 25–30 min design session commenced, yet participants had the flexibility to end their design activities earlier or later based on their design progress. All pairs of designers were required to develop at least one final deliverable at the end of the design session. After a five-minute break, participants were placed in separate rooms without any communication before task 2 commenced (see Figure 2). Participants were instructed to join a ZOOM meeting and enabled their camera and microphone for virtual communication. They were also asked to change their displayed names to their assigned identification numbers. The design requirement for task 2 is to cooperate on a conceptual design for multi-functional furniture that could be used indoors and outdoors. Repeating the same steps of task 1, dyads were told to undertake a five-minute virtual warm-up session for design brief exploration and another 25–30 min design session employing the whiteboard feature in the ZOOM meeting, and participants were also allowed to end the design activities earlier or later accordingly. Figure 3 well illustrates the designated experimental sequences and time frames.

**Figure 4:**
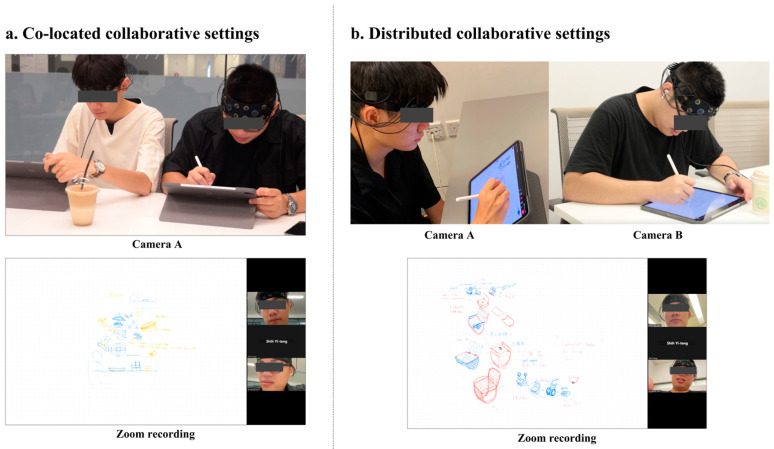
(**a**) Zoom recording and one camera’s fields of view for co-located collaboration contexts. (**b**) Zoom recording and another two cameras’ fields of view for distributed collaboration environment.

In this study, digital sketching using the ZOOM whiteboard feature was utilized in both collaborative contexts (co-located and distributed), aiming to eliminate the influence of different sketching tools (pen-and-paper sketching and digital sketching) on the design outputs [46,47]. In order to record participants’ design activities and interactions, ZOOM recording was conducted while completing design sessions for capturing verbalization and sketching activities. In addition, other video cameras were installed in front of each dyad for identifying non-verbal design behaviors and interactions, such as eye contact and body language. Figure 4 demonstrates specific cameras’ fields of view.

### 3.3. Instruments and Computational Tools

**Figure 5:**
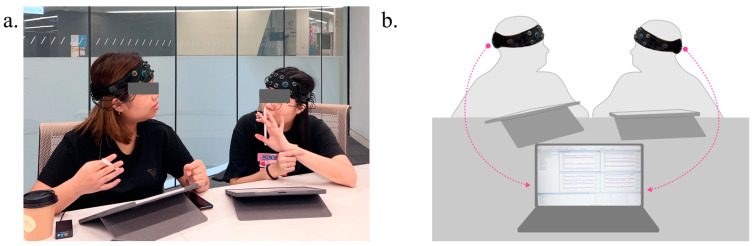
(**a**) fNIRS headcap settings for data collection during co-located design collaboration; (**b**) scanning data were recorded through OxySoft 3D software, supporting the recording of two individuals’ brain activities on one laptop concurrently.

**Figure 6:**
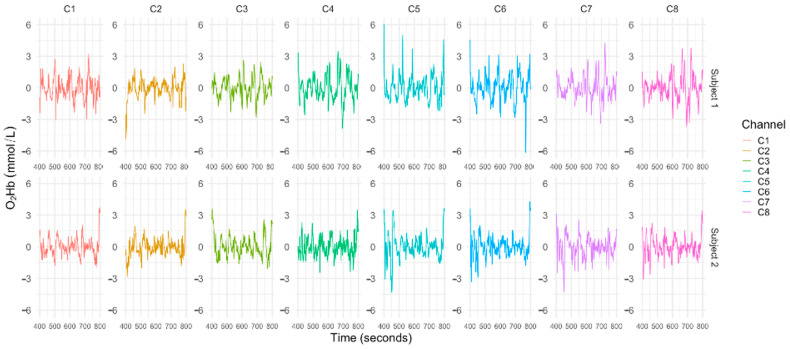
Example of fNIRS data collected from one of the dyads. Notes: due to the length and drifting of the signal, the signal presented above was extracted from one of the dyads with a time window of 400–800 s.

One prominent technique used in hyper-scanning research is fNIRS, a non-invasive neuroimaging method that utilizes near-infrared light to penetrate the scalp and skull, enabling the monitoring of hemodynamic responses in specific brain regions. Correlative neurocognition reviews [30,48] have highlighted the wide use of fNIRS in investigations focusing on brain-to-brain communication during social interaction tasks, especially during interpersonal cooperation. fNIRS has been used as a dominant complement to fMRI and EEG to measure IBS, as its reasonable spatial resolution, greater resilience to body movement, and less experimental settings limits frequently applied for IBS measurement within naturalistic interactive environments. Therefore, we conducted a fNIRS-based hyper-scanning study. Each participant was fitted with fNIRS (OctaMon, Artinis Medical Systems, the Netherlands, as shown in Figure 5a) headcap on the forehead for the assessment of cerebral blood flow (CBF) changes in the prefrontal cortex. Scanning data were recorded using OxySoft 3D software 4.0.6.1 x64, which supports recording two individuals’ changes in oxy-hemoglobin (HbO) and deoxy-hemoglobin (HbR) levels on one laptop simultaneously in both co-present and distributed settings. Figure 6 presents the instance of the signal extracted from one of the dyads with a time window of 400–800 s.

All signal processing and statistical analyses were performed using the R programming (Version 4.3.1) language, a powerful tool for statistical computing and graphics. To facilitate these computations, we utilized a variety of relevant R packages designed for data manipulation, signal processing, and statistical modeling. These tools allowed us to process the fNIRS data, perform the necessary statistical tests, and derive meaningful results about the patterns of IBS under different conditions and behaviors in the context of design collaborations.

### 3.4. Data Analysis

#### 3.4.1. Interaction Segmentation

The video recording data were initially filtered by coding for off-task behaviors (e.g., jokes, banter between the designers, and conversation of events unrelated to the design problem). Subsequently, the video data was segmented into small episodes and coded for three design interaction behaviors: verbal-only, sketch-only, and mixed communication (a combination of verbal and sketch). Verbal-only means that within a certain period, the design ideas are only proposed and transmitted through verbal communication. Similarly, sketch-only means that only sketch activities take place during a certain period of time, as the only methods of conceptual exchange. In terms of mixed communication, which means that verbalization and sketching occur simultaneously during a period or quickly alternate. Table 1 presents specific instances collected from one of the dyads, elaborating on each defined design behavior observed during the design collaboration process. Two well-trained investigators conducted the episode segmentation separately aligning with the same criteria. By comparing the segments classified by the 2 investigators, 23 controversial segments were excluded. Overall, 432 segments were extracted, of which 250 episodes occurred within the co-located context and 182 were distributed collaborations.

#### 3.4.2. IBS Analysis

##### fNIRS Data Segmentation and Variance Estimation

The continuous fNIRS data were divided into 15 s windows according to those behavior segments defined in Table 1. For each of these time windows and each channel, the variance was calculated. To ensure the quality of the signals and eliminate outliers, time windows with variance values above or below 2.5 standard deviations from the mean variance calculated across all time windows and channels were excluded. This step was crucial to filter out periods with potential motion artifacts, which could result in high variance, and dead periods (where no signal was captured), which would result in low variance. Since the behaviors of each pair of participants varied (i.e., some pairs may exhibit more verbal behavior, while others may exhibit more sketching behavior), the selected time windows were randomly sampled to avoid bias. For each condition (face to face and remote) and each behavior (verbal only, sketch only, and simultaneous verbal and sketch), 10 times windows were selected.

##### Establishment of Synchrony Measurement

The fNIRS device used in this study was capable of capturing data from eight channels per participant. Four of these channels were situated on the left frontal region of the brain and four on the right frontal region, thereby allowing for a comprehensive capture of brain activity across these vital areas. After the data collection, the channels were aggregated for each side of the brain for each participant. This process resulted in four distinct data sets: Participant 1’s left frontal activity, Participant 1’s right frontal activity, Participant 2’s left frontal activity, and Participant 2’s right frontal activity. Synchrony measures were then established around the two regions of interest—left and right frontal regions—for each participant. This resulted in four distinct paths for IBS analysis: (i) Participant 1 Left Frontal to Participant 2 Left Frontal; (ii) Participant 1 Left Frontal to Participant 2 Right Frontal; (iii) Participant 1 Right Frontal to Participant 2 Left Frontal; (iv) Participant 1 Right Frontal to Participant 2 Right Frontal. These paths provide a comprehensive framework for analyzing the interplay and synchrony of brain activities between the participants during their design collaboration under various conditions and modes of communication.

**Figure 7:**
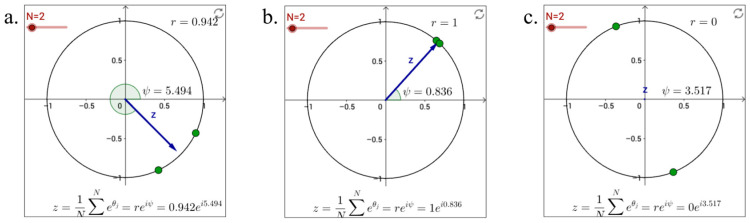
Illustration of Kuramoto Order Parameter (the phase angles for two signal sources are depicted by green dots, originating from their respective unit vectors; the blue arrows represent the mean direction of these unit vectors). (**a**) the proximity of phase angles between the two signals results in a magnitude nearly equal to 1; (**b**) shows that the phase angles from both sources are aligned, producing a magnitude of exactly 1; (**c**) illustrates that the sources are in antiphase, leading to a magnitude of 0.

IBS between any pair of sites were measured with the average length of the Kuramoto Order Parameter (KOP). The KOP measures the phase synchrony between two signals by calculating the vector average of phase angles over time (see Figure 7). A value of 1 indicates perfect phase synchrony, while a value near 0 indicates no phase synchrony. The KOP was calculated for each pair of brain sites in each experimental condition. The average KOP length across time was then used as the index of IBS between those two sites. Higher average KOP lengths indicate greater IBS.

##### IBS Calculation

To calculate IBS, we extracted 20 non-overlapping 15 s time windows from the brain signal data timed to the interaction between the two participants as shown in the video. Within each window, we calculated the instantaneous Kuramoto Order Parameter (KOP), denoted *r*, between the two brain sites at each time point. For example, with 150 time points, this gave 150 *r* values. We averaged these *r* values to obtain a “window-level” synchrony value representing the phase synchrony during that 15 s period. We then averaged the 20 “window-level” *r* values within each experimental condition to obtain a “dyad-level” mean KOP, denoted $\bar{r}$, for that condition. Since there were 20 window-level values per condition, each dyad-level $\bar{r}$ represented the mean synchrony across those 20 time periods. The synchrony index $\bar{r}$ ranges from 0 to 1. A value of 0 indicates completely out-of-phase signals, while 1 indicates perfect in-phase synchrony. Intermediate $\bar{r}$ values indicate partial synchrony. Thus, the dyad-level $\bar{r}$ reflected the average phase synchrony between the two brain sites during a given experimental condition.

## 4. Results

### 4.1. IBS Analysis

To understand the effects of different conditions (co-located vs. distancing) and behaviors (verbal only, sketch only, mixed interaction (verbal + sketch)), we employed a linear mixed model. This model allowed us to establish the main effects of condition and behavior, as well as their interaction effects. Following this, post hoc pairwise comparisons were calculated where appropriate to further delve into the differences between the conditions and behaviors in terms of their influence on IBS. Given the small sample size of this preliminary study, we refrained from reporting the statistical results at the individual channel-to-channel synchrony level. However, to provide insights into the patterns of IBS, we still present the mean values of synchrony for each of the four established paths: (i) Participant 1 Left Frontal to Participant 2 Left Frontal; (ii) Participant 1 Left Frontal to Participant 2 Right Frontal; (iii) Participant 1 Right Frontal to Participant 2 Left Frontal; and (iv) Participant 1 Right Frontal to Participant 2 Right Frontal. These mean values offer a preliminary view of the patterns of IBS under different conditions and behaviors, further contributing to our understanding of collaborative cognition in design tasks.

**Figure 8:**
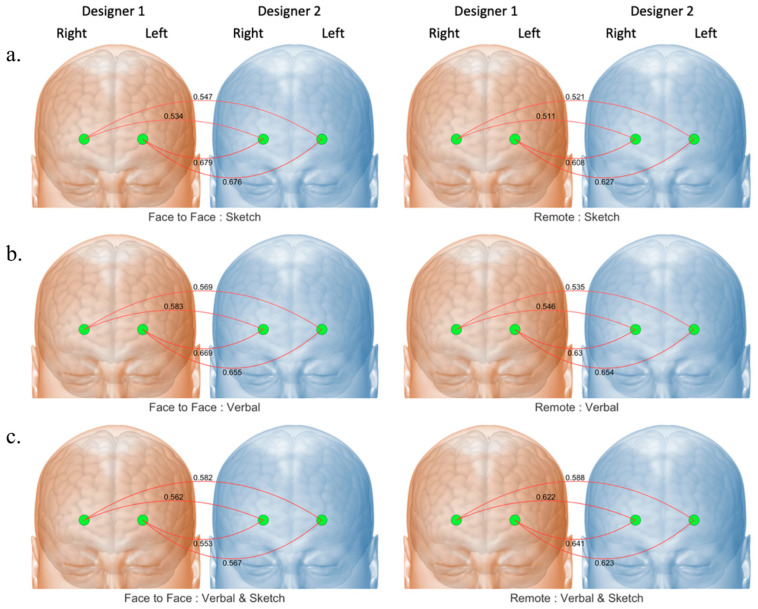
Average IBS by condition, behavior, and inter-brain connectivity pathways. (**a**) the IBS of the first dyad (female-female); (**b**) the IBS of the second dyad (male-male); (**c**) the IBS of the third dyad(female-male).

Our preliminary study revealed several findings that highlight the differences in IBS among designers collaborating in a face-to-face (F2F) setting compared with a remote setting (see Figure 8). The interactions were categorized into three design behaviors: verbal communication only, sketching only, and mixed communication (V + S). The IBS was higher during the sketch-only behavior in the F2F condition compared to the remote condition. Conversely, during the V + S behavior, the IBS was higher in the remote condition than in the F2F condition. In addition, in the F2F condition, IBS was greater during the sketch-only behavior than during the V + S behavior. In the remote condition, however, the IBS was higher during the V + S behavior than during the sketch-only behavior. In the F2F condition, the IBS was smallest during the V + S behavior compared to both verbal-only and sketch-only behaviors.

### 4.2. Statistical Analysis

To examine the influence of experimental conditions and behaviors on inter-brain synchrony (IBS), we specified a linear mixed-effects model with the formula IBS~Condition × Behavior + (1|Dyad) + (1|Path). In this model, IBS is the dependent variable, reflecting the inter-brain synchrony coefficient. Condition and behavior are treated as fixed effects, with their interaction term, Condition × Behavior, investigating whether the effect of one factor is contingent on the level of the other. Random effects terms (1|Dyad) and (1|Path) introduce random intercepts for each dyad and each path, respectively, which allow for the modeling of variability within dyads and paths that are not captured by the fixed effects.

**Figure 9:**
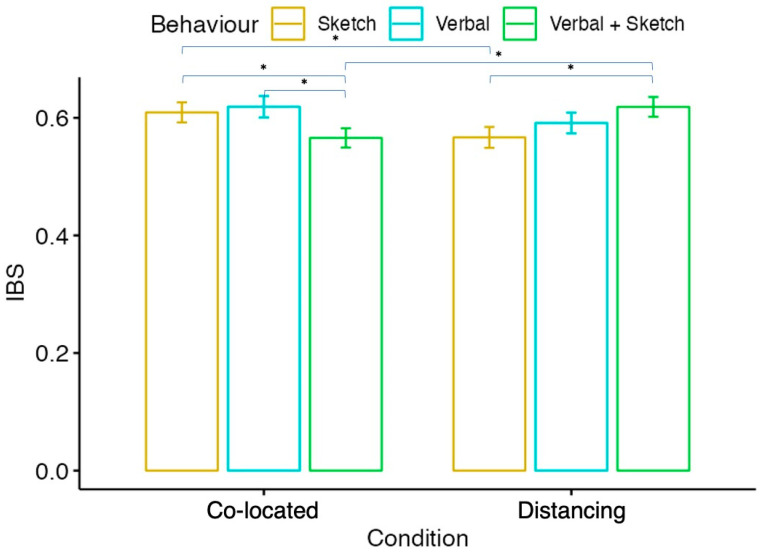
IBS by condition and behavior. Note: Asterisks (*) denote statistical significance with a *p*-value less than 0.05.

**Figure 10:**
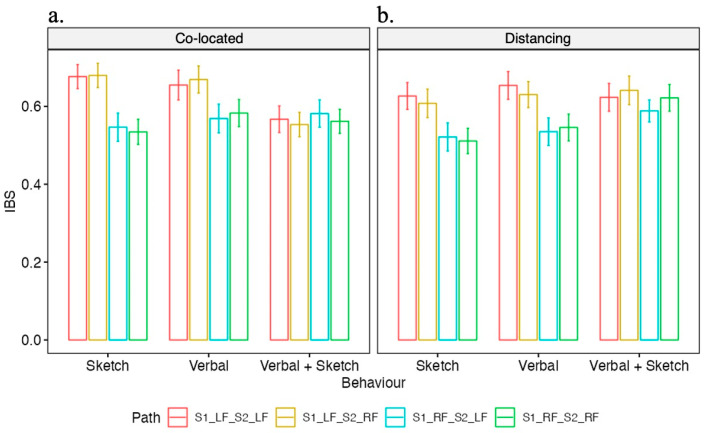
IBS by condition, behavior and inter-brain connectivity pathways (Note—S1_LF_S2_LF: Designer 1 Left Frontal to Design 2 Left Frontal; S1_LF_S2_RF: Designer 1 Left Frontal to Design 2 Right Frontal; S1_RF_S2_LF: Designer 1 Right Frontal to Design 2 Left Frontal; and S1_RF_S2_RF: Designer 1 Right Frontal to Design 2 Right Frontal). (**a**) illustrates the Inter-Brain Synchrony (IBS) occurring in a co-located setting, where two designers are seated together, engaging in face-to-face communication during the design discussion; (**b**) illustrates the distancing condition, the designers are situated in separate rooms, with communication enabled through video conferencing software that allows screen sharing and collaborative drawing.

Model fitting was conducted using the ‘lme4’ package in R. To determine the significance of the fixed effects, we performed an Analysis of Variance (ANOVA) using the ANOVA function from the stats package, which yielded an ANOVA table presenting the degrees of freedom, the sum of squares, mean squares, F-statistics, and associated *p*-values for each fixed effect (see Table 2). To ascertain the necessity of including random effects in our model, we compared it to simpler nested models with different random effects structures. Specifically, we compared our full model (IBS~Condition × Behavior + (1|Dyad) + (1|Path)) to a model with random intercepts for dyads only (IBS~Condition Behavior + (1|Dyad)) and to a linear model without random effects (IBS~Condition × Behavior). These comparisons were made using chi-square tests (Figure 9 and Figure 10), which are appropriate for comparing nested models differing in complexity. Additionally, the chi-square test results indicated that the full model with both (1|Dyad) and (1|Path) random effects provided a significantly better fit to the data than the models with fewer random effects, justifying the inclusion of both random intercepts in our analysis. For post hoc analysis (see Table 3), pairwise comparisons of the estimated marginal means for each pair of conditions within behavior levels and each pair of behaviors within condition levels were executed using the ‘emmeans’ package. We addressed the multiple comparison issue by applying the False Discovery Rate (FDR) correction using the Benjamini–Hochberg procedure to control for type I errors.

### 4.3. Design Outcome Evaluation

The three design teams were able to satisfy both toy design and multifunctional chair tasks between co-located and distributed modes. Table 4 shows the evaluation results of the digital sketches by the four design experts. The scores in Table 5 are the average scores of the four reviewers. The fourth column (average score) in each mode shows the average performance of the three design teams for each criterion.

Team 1’s co-located mode design outcome received higher scores in terms of satisfying the design task (7.1 vs. 6.6) and practical solution (7.3 vs. 6.4). The criterion of flexibility of the design score was closer for the two design outcomes (6.2 vs. 6.3). In addition, the average scores for both collaboration modes were very similar for Team 2 and Team 3 (6.2 vs. 6.1 and 6.0 vs. 6.0). Overall, the three design teams in both collaboration modes produced very similar design outcomes.

## 5. Discussions

Our study provides valuable insights into the nuances of collaborative design processes under different conditions, and how these conditions can influence IBS. The increased IBS during the sketch-only behavior in the F2F setting suggests that co-located interactions might facilitate a better shared understanding when designers are expressing their ideas purely through sketches. This could be attributed to the immediate and unfiltered feedback made possible by real-time, physical interactions. In this setting, non-verbal cues such as body language or facial expression might also play a significant role. In contrast, the higher IBS observed during the mixed communication (verbal and sketch) in the distributed condition indicates that this combination of communication modes might be more effective in synchronizing the designers’ cognitive processes when working remotely. This might be due to the increased reliance on verbal communication to form a shared understanding in the absence of physical presence. The need to articulate thoughts clearly and concisely during remote collaboration might lead to a more coordinated cognitive process.

Interestingly, IBS was greater during sketch-only behavior compared to mixed verbal and sketching communication in the co-located condition. Conversely, within the remote collaboration setting, IBS was elevated during mixed communication versus sketch-only behavior. This suggests the form of interaction that best facilitates cognitive alignment may heavily depend on whether design partners share physical space. Notably, the lowest IBS in face-to-face collaboration occurred with simultaneous verbalization and sketching, potentially indicating heightened cognitive demands that desynchronize neural processes. The richness of contextual cues in physical proximity may further challenge integration across multiple communication modes. Furthermore, the highest IBS took place when collaborators communicated in verbal-only within the co-located condition, while the mixed communication (verbal and sketch) behavior promoted the highest IBS during online design collaboration. This finding supports the prior discoveries [29] of increased time allocated to sketching in virtual teams. These preliminary findings could profoundly influence the development of optimally collaborative work environments and digital platforms. Further research should elucidate the precise mechanisms relating design team synchrony to performance across contexts, providing actionable direction for enhancing collective innovation.

The limitation of this preliminary study constrains the generalizability of the findings and conclusions. Firstly, the small sample size of the three dyads restricts the statistical power and precludes drawing definitive conclusions regarding the impact of different collaborative settings on IBS patterns and design outcomes. Additional participants are necessary to quantitatively discern such effects. Additionally, the limited age range and design experience level of the participants may not represent the true diversity of collaborative teams in real-world design practices. Broader sampling would augment ecological validity. Therefore, future studies will be conducted on larger and more diverse samples, considering additional confounding variables, such as gender, age ranges, and years of design experience.

## 6. Conclusions

This study investigated the patterns of brain synchrony among design collaborators during the conceptual design process within two collaborative environments: distributed and co-located settings. The consequences based on the preliminary study emphasize several variations in IBS among designers collaborating in these two settings. Through protocol analysis, interactions between each dyad were classified into three categories: verbal-only, sketch-only, and mixed interaction (verbal and sketch). Subsequently, according to the hyper-scanning analysis, the increased IBS was observed during the sketch-only behavior in the co-located setting, suggesting that sketching might be a facilitator for better mutual understanding when design collaboration occurs face to face. This could be attributed to the immediate and unfiltered feedback made possible by real-time, physical interactions. Comparably, our results revealed a higher level of IBS when subjects employed mixed communication (verbal and sketch) in distributed conditions, demonstrating the combination of verbal communication and sketching might lead to a more coordinated cognitive process when physical isolation.

Moreover, the IBS was greater during the sketch-only behavior than during the mixed communication behavior within the co-present setting. Interestingly, the level of IBS was found to be higher when participants performed sketch-only behavior compared to mixed communication behavior in the co-present settings, while the IBS was higher during the combination of verbal and sketching behavior within remote settings. This finding illustrates the close associations between the utilization of communication methods improving cognitive synchrony and collaborative environments. Design collaborators are inclined to adjust their interaction behaviors in order to adapt to different design environments and strengthen the exchange of opinions and the construction of consensus. Furthermore, the results indicate that there were no significant differences in overall collective performance and design outputs between these two collaboration contexts.

To draw statistical conclusions on the impact of IBS on team behavior and performance, it is suggested that future studies be conducted with a larger sample size along the same framework. The preliminary study demonstrated how neuroimaging can be used to analyze behavioral patterns in two different collaboration environments. It could be a step towards building effective virtual teamwork beyond the design realm. Furthermore, these findings could have important implications for the design of collaborative workspaces with digital tools. Further study is needed to better understand the underlying mechanisms and how these insights could be applied to optimize team performance in design contexts. In subsequent research, interventions that promote IBS can be tested, such as team training, introducing diversity within groups, and assessing their impact on IBS.

## Figures and Tables

**Table 1:** Examples of detailed transcripts and sketching activities (screenshots) in each design condition: verbal only, sketch only, and mixed communication (sketch and verbal).

No.	Condition	Specific Behaviour	Instances (Scripts and Screenshots)
1	Verbal only	Participant A explained ideas by verbalization only. Participant B plays as an audience.	A: “What do you think about this, like this, stacking all the modules.” A: “It’s a bit like Noah’s Ark. Or it can also be stacked like a pyramid.” B: “I think it looks great.”
		Participant B explained ideas by verbalization only. Participant A plays as an audience.	B: “I’m thinking we can keep the under layer as a circle shape.” B: “Because if the shape is too flat, it may not float on the water, or maybe it could be just like a little boat.” A: “I can picture what you are describing.”
		Participants exchange design ideas alternately and finally reach a consensus.	A: “But the size of this ball cannot be too small” B: “But if the shape is a ball, they might fall easily” A: “I think it’s okay, because they can float on the water” B: “Oh! You’re right.”
2	Sketch only	Participant A explains ideas by sketching only. Participant B plays as an information receiver.	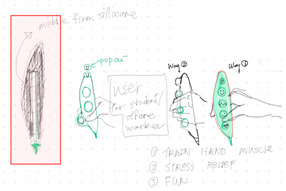
		Participant B explains ideas by sketching only. Participant A plays as an information receiver.	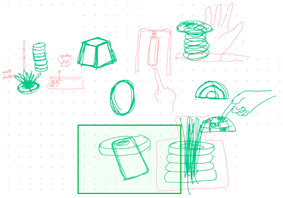
		Participants design by sketching collaboratively.	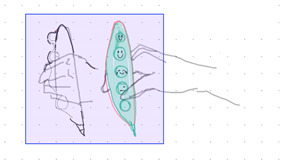
3	Mixed communication (Sketch + Verbal)	Participant A is information provided by think aloud and sketching concurrently. Participant B plays as an information receiver.	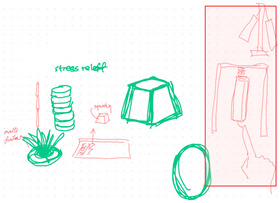
		Participant B is information provided by think aloud and sketching concurrently. Participant A plays as an information receiver.	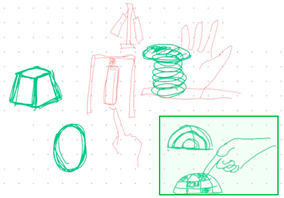
		Participants A and B think aloud and sketch collaboratively.	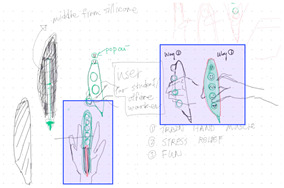

**Table 2:** ANOVA test for the interaction effect between condition and behavior.

Variable	Sum Sq	Mean Sq	NumDF	DenDF	F Value	*p*-Value
Condition	0.00901	0.009011	1	1073.8	0.172	0.678
Behavior	0.05676	0.028382	2	1073.8	0.5417	0.582
Interaction	0.47281	0.236405	2	1073.8	4.5124	0.011

Notes: Sum Sq: Sum of Square; Mean Sq: Mean Sum of Square; numDF = numerator degrees of freedom; denDF = denominator degrees of freedom.

**Table 3:** Post hoc analysis of pairwise comparisons at each level of condition and behavior.

Condition	Behavior	Contrast	Estimate	SE	df	T Ratio	*p*-Value	fdr *p*
F2F	/	Sk − Vb	−0.010	0.024	1082.02	−0.393	0.691	0.691
F2F	/	Sk − VS	0.043	0.024	1082.02	1.775	0.073	0.143
F2F	/	Vb − VS	0.053	0.024	1082.02	2.168	0.029	0.097
Remote	/	Sk − Vb	−0.024	0.024	1082.02	−1.001	0.311	0.350
Remote	/	Sk − VS	−0.052	0.024	1082.02	−2.120	0.032	0.097
Remote	/	Vb − VS	−0.027	0.024	1082.02	−1.119	0.258	0.332
**/**	Sk	F2F − remote	0.042	0.024	1082.02	1.737	0.079	0.143
**/**	Vb	F2F − remote	0.028	0.024	1082.02	1.129	0.254	0.332
**/**	VS	F2F − remote	−0.053	0.024	1082.02	−2.158	0.029	0.097

Notes: Sk = sketch only; Vb = verbal only; VS = verbalization + sketch; F2F: co-located interaction; Remote: distancing interaction.

**Table 4:** Screenshot of the final design solutions by digital sketches of each team within different collaborative environments.

	Co-Located Mode	Distributed Mode
Team 1	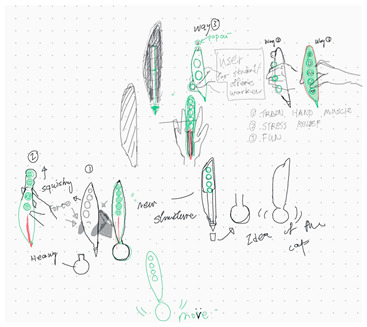 An office toy used for hand relaxing	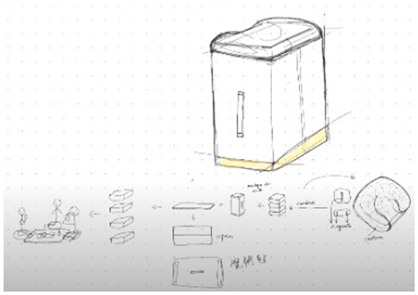 A versatile chair can be used as a furniture stool at home or as a picnic blanket outdoors.
Team 2	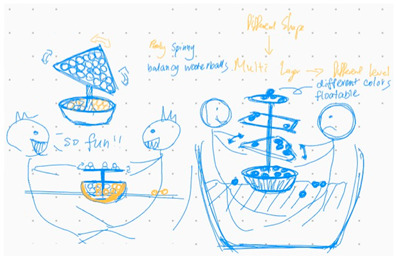 A block toy to play with during the bath, designed for kids aged ranged between 3-6 years.	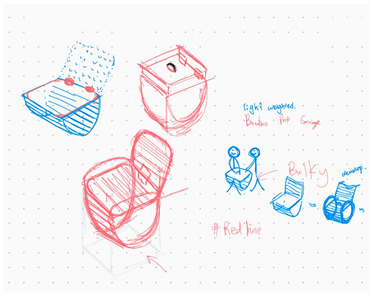 A multifunctional DIY outdoor chair for elderly.
Team 3	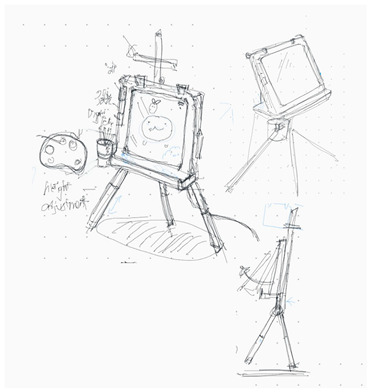 A multifunctional magic drawing board for early learning.	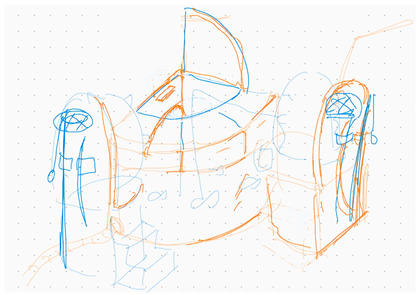 A multifunctional dining table designed for two cartoon characters.

**Table 5:** Scores for the design outcomes within different design conditions.

Criteria	Co-Located Mode				Distributed Mode			
	Team 1	Team 2	Team 3	Av	Team 1	Team 2	Team 3	Av
How innovative	6.1	5.5	4.8	5.5	5.3	5.1	4.5	5.0
How creative	6.3	6.0	5.3	5.9	5.8	5.6	6.1	5.8
Satisfying design task	7.1	6.7	6.0	6.6	6.2	6.6	6.8	6.5
Practical solution	7.3	6.5	6.7	6.8	6.0	6.4	5.7	6.0
Flexibility of the design	6.2	6.4	7.1	6.6	6.3	6.6	7.0	6.6
Av	6.6	6.2	6.0	6.3	5.9	6.1	6.0	6.0

## Data Availability

The datasets generated and/or analyzed during the current study are not publicly available due to privacy but are available from the corresponding author upon reasonable request.
